# Effect of Ultrasound Time on Structural and Gelling Properties of Pea, Lupin, and Rice Proteins

**DOI:** 10.3390/gels11040270

**Published:** 2025-04-04

**Authors:** Natalia Riquelme, Paulo Díaz-Calderón, Alejandro Luarte, Carla Arancibia

**Affiliations:** 1 Laboratorio de Investigación en Propiedades de los Alimentos (INPROAL), Departamento de Ciencia y Tecnología de los Alimentos, Facultad Tecnológica, Universidad de Santiago de Chile, Estación Central 9170201, Chile; natalia.riquelme.h@usach.cl; 2 Biopolymer Research & Engineering Laboratory (BIOPREL), Escuela de Nutrición y Dietética, Facultad de Medicina, Universidad de Los Andes, Chile, Las Condes 7620001, Chile; pdiaz@uandes.cl; 3 Centro de Investigación e Innovación Biomédica (CIIB), Universidad de Los Andes, Chile, Las Condes 7620001, Chile; 4 Facultad de Medicina, Universidad de Los Andes, Chile, Las Condes 7620001, Chile; aluarte@uandes.cl; 5 Programa de Neurociencias, Centro de Investigación e Innovación Biomédica (CIIB), Universidad de Los Andes, Chile, Las Condes 7620001, Chile

**Keywords:** plant proteins, ultrasound treatment, structural and techno-functionality characteristics, gelling properties

## Abstract

Plant proteins are garnering interest due to the growing demand for plant-based products, but their functionality in gel-based foods remains limited. Ultrasound (US) technology may improve the technological properties of proteins. Thus, the effect of US treatment time (0–15 min) on the structure and gelling properties of pea, lupin, and rice proteins was evaluated. The results showed that the whiteness (~60%) of all freeze-dried proteins remained unchanged (*p >* 0.05), regardless of the US time. However, FT-IR analysis revealed progressive reductions in α-helix and β-sheet for pea and lupin proteins (~50%) with US time, indicating partial unfolding. In addition, microstructure analysis showed an ~80% reduction in aggregate size for these proteins, while rice protein exhibited minimal changes. Conversely, weak gels were formed with pea and lupin proteins treated after 5 and 10 min of US, respectively, whereas rice protein did not form gels. Furthermore, US treatment time significantly increased (*p <* 0.05) the mechanical moduli, resulting in more structured gels after longer treatment times (tan δ ~0.3 at 15 min of US). These findings suggest that US treatment enhances the gelling properties of pea and lupin proteins, making them more suitable for plant-based food applications such as yogurt or desserts.

## 1. Introduction

The global population’s dietary habits have changed, with an increase in the consumption of plant-based food products, particularly plant proteins [1]. These proteins have a greater potential to promote consumer health and are more environmentally friendly and sustainable than animal sources [2,3]. The food industry faces a significant challenge in providing healthy plant-protein-based foods that meet the requirements of consumers who have modified their diets, such as vegans, vegetarians, and flexitarians [4].

The main problems of plant proteins are their lower technological functionality, such as hydration [5], solubility [6], gelling [7], and emulsifying properties [8]. These limitations restrict their application in developing plant-based analogs [9,10]. Plant proteins differ in their polypeptide sequences and the proportions of their secondary and tertiary structures [5], which can alter their molecular and functional properties [11]. Additionally, plant proteins exhibit structural differences compared to animal proteins, which affect their functional characteristics [12]. For instance, plant proteins tend to have larger and more compact structures, while muscle proteins have flexible and fibrous conformations. This compactness can negatively impact the overall techno-functional properties of plant proteins [13]. In this context, enhancing plant protein functionality using emerging processing technologies can help to develop better-quality plant-based foods, where understanding how process conditions can modify the structure and the technological properties of such proteins is critical for designing new gel-like analogs.

Among plant proteins, pea protein has become popular in the plant-based industry for the preparation of meat and dairy beverages [14,15,16] due to its low allergenicity, high nutritional value, wide availability, non-GMO status, and low cost [17,18]. Other protein sources like rice and lupin have also gained attention for their potential use in plant-based foods. Rice protein is a promising option due to its high quality, since it has a balanced amino acid ratio and hypoallergenic characteristics [19,20,21]. Some studies have also suggested that rice protein can promote health benefits, such as antihypertensive, antioxidant, and anticarcinogenic activities [22]. Meanwhile, lupin protein is a new and sustainable source of plant protein which is cheaper than soybean proteins [23,24,25]. Furthermore, this legume has a high protein content (~40%) and low anti-nutrient components, making it suitable for plant-based dairy products [26]. In this sense, lupine protein isolates from the species *Lupinus luteus* (AluProt-CGNA^®^) contain a higher amount of protein (~60%) compared to other legumes commonly used in the food industry [27,28]. However, these plant proteins have a poor aqueous solubility, which can impact other functional properties of proteins and their food processing [18]. Therefore, research is needed on these three plant proteins to enhance their technological properties, offering new applications for the development of plant-based foods.

Among the traditional processes used to enhance the technological properties of proteins, heat treatment stands out as one of the most commonly employed. Controlled heat processes can alter protein structures, thereby improving certain functional properties. However, heat treatment also leads to irreversible protein unfolding, resulting in the formation of new molecular arrangements, which can cause the opposite effect [29]. In addition, these conventional treatments are highly aggressive and compromise the nutritional and sensory profile of proteins [30]. For this reason, non-thermal processes (such as extrusion, pulsed electromagnetic fields, supercritical fluid extraction, high pressure, and ultrasound) have emerged as promising methods for protein modification. These processes offer minimal nutrient loss, enhance food safety, and improve energy efficiency, making them a feasible alternative to replace traditional thermal treatments [31]. Among these non-thermal treatments, ultrasound (US) has gained particular attention due to its nontoxic nature, cleanliness, and absence of solvents, being an environmentally friendly option [32,33]. Moreover, ultrasound-induced cavitation can cause changes in the physical characteristics of proteins by effects of both denaturation and aggregation. These changes are due to the destruction of chemical bonds that maintain the protein structure (such as hydrogen bonds, ionic bonds, and hydrophobic interactions), which results in a loose and stretched (unfolded) protein structure [34]. In addition, this process changes the particle size, microstructure, electrical charge and conductivity, and rheological properties [35], which has shown improvements in techno-functional properties such as emulsifying and gelling capacity [36].

In this sense, several studies have reported positive effects of US treatment on the functionality of plant proteins, such as the emulsifying properties of soy protein [37], the solubility and foaming capacity of faba bean protein [38], and the emulsifying and gelling properties of insoluble potato protein isolates [39]. However, there is limited research on pea, rice, and lupin proteins, highlighting the need for further studies to better understand the changes induced by ultrasound (US) treatment on the techno-functional properties of these and other plant protein sources. Therefore, this study will analyze the impact of US treatment (with different process times of 5, 10, and 15 min) on the structural changes and gelling properties of pea, lupin, and rice proteins, aiming to improve their behavior during plant-based gel preparation. This research aims to bridge the existing knowledge gap and provide insights into optimizing protein processing with ultrasound. Additionally, this research provides valuable insights for the food industry, particularly for manufacturers developing plant-based yogurts, desserts, and meat analogs, by the application of US treatment to enhance protein functionality.

## 2. Results and Discussion

### 2.1. Characterization of Freeze-Dried Plant Protein

#### 2.1.1. Optical Properties

**Figure 1:**
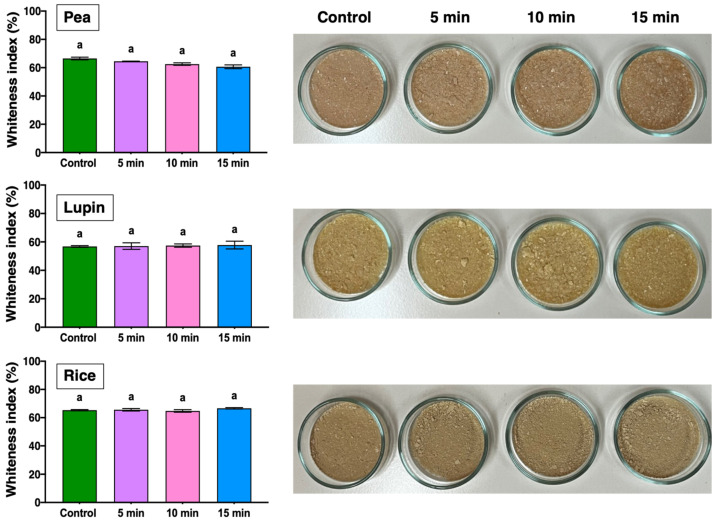
Whiteness index of different freeze-dried plant proteins treated at different times of ultrasound. Different letters indicate significant differences (*p <* 0.05) between means for US treatment times.

Optical properties are important because they significantly affect consumers’ acceptance of food products elaborated from plant proteins. Therefore, the impact of US treatment on the optical properties of plant proteins was assessed by determining their whiteness index (WI). Figure 1 presents the WI values for the plant proteins, which are approximately 60%.

Notably, all samples exhibited a brown color, showing minimal differences among the different types of proteins. Lupine protein presented WI values ranging from 56.8 to 57.8%, whereas pea and rice proteins showed higher values between 60.7 and 66.5% and 64.8 and 66.6%, respectively. These differences are also evident in photographs of the proteins, where lupine exhibits a more yellow color than the pea and rice samples. In addition, the differences in the WI values among the proteins might have been due to their source. On the other hand, US treatment did not influence the WI values for any of the plant proteins, as reflected in the photographs of the samples. This result is relevant for the food industry, since maintaining the whiteness of plant proteins is crucial for their visual appeal and consumer acceptance.

#### 2.1.2. FT-IR Spectra and Changes in the Secondary Structure of Proteins

**Figure 2:**
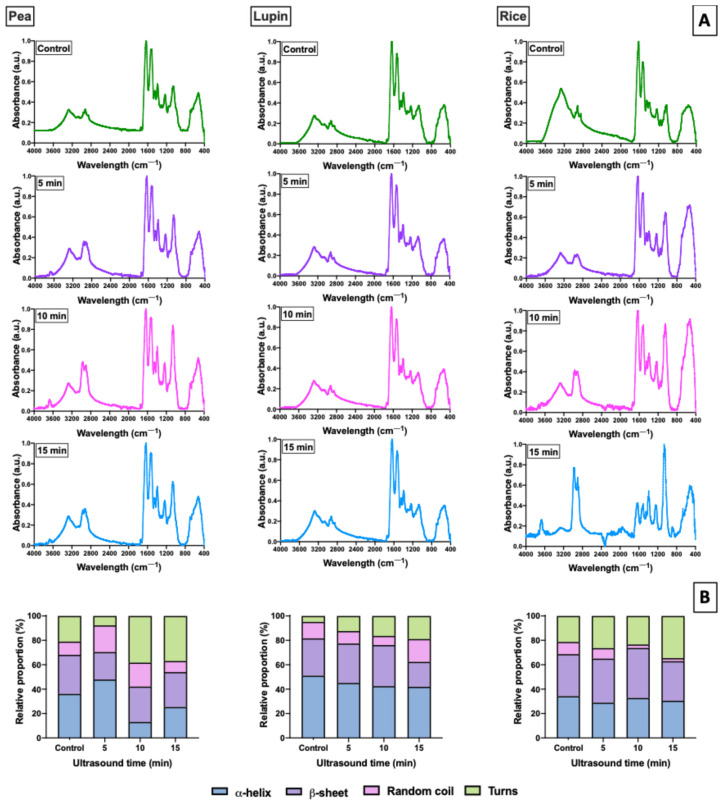
FT-IR spectra of different freeze-dried plant proteins treated with different times of ultrasound (**A**) and its relative content of the components of the secondary structure (**B**).

The FT-IR spectra of plant proteins treated with ultrasound (US) are shown in Figure 2A. As expected, characteristic absorption bands corresponding to the amide I region (1700–1600 cm^−1^) were observed in all samples. This region is associated with the C=O stretching vibration of the amide group in proteins [40]. The main band of the amide I region appeared near 1630, 1635, and 1625 cm^−1^ for pea, lupin, and rice proteins, respectively (Figure 2A). Also, a slight shift was observed with an increasing US treatment time (shifting to 1625, 1630, and 1620 cm^−1^ after 15 min of US). Similarly, absorption bands around 1600–1500 cm^−1^ were also observed, which are associated with the amide II region, corresponding with the C–N stretching and N–H bending of proteins [41]. Furthermore, differences in the amide bands’ intensities were detected, suggesting that US treatment altered the secondary structures of the samples, with these changes being dependent on the US time.

For this reason, the modification in the secondary structures of the proteins treated with US was evaluated as the changes in the band positions around amide I. According to Shevkani et al. [42], bands corresponding to the amide I region confirm the strong presence of α-helix (1650–1660 cm^−1^), β-sheet (1630–1638 cm^−1^), β-turn (1660–1680 cm^−1^), and random coil (1643–1645 cm^−1^) conformations of proteins. As shown in Figure 2B, the relative content of the secondary structure in the different proteins changed with an increasing US treatment time. In this sense, α-helix and β-sheet were predominant in the native plant proteins (control samples without US treatment), with the relative content of α-helix being 36.04, 51.00, and 32.23% and that of β-sheet being 32.12, 30.63, and 34.46% for pea, lupin, and rice control samples, respectively. However, the α-helix and β-sheet contents of pea and lupin proteins decreased after different US times, mainly after 15 min. This behavior varied depending on the protein type and secondary structure. In the case of pea protein, the reduction in α-helix content was more pronounced, with values dropping from 36.04% (control sample) to 25.32% after 15 min of US treatment. Meanwhile, the β-sheet content showed the most significant reduction in lupin protein, with a decrease of 33.37%. In addition, a gradual increase in random coil and β-turn content was observed in pea and lupin proteins (Figure 2B). This increase was most evident in the β-turn secondary structure, showing a rise of 176.22% in pea protein and 389.71% in lupin protein. So, our results suggest that the cavitation destroyed the hydrogen bonds between the carbonyl group and the amino group on the polypeptide chain, stabilizing the α-helix and β-sheet structures [40], producing a decrease in the content of these structures (α-helix + β-sheet) and an increase in β-turn and random coil structures. 

In rice protein, its secondary structure content showed only slight variations after US treatment. The relative content changed as follows: α-helix from 34.23% to 30.49%, β-sheet from 34.46% to 32.43%, random coil from 10.05% to 2.49%, and β-turn from 21.26% to 34.59% for the control and 15 min of US, respectively (Figure 2B). These small changes observed in the rice protein’s secondary structure could indicate its low sensitivity to the US treatment, where the applied US energy was not high enough to change the secondary structure of rice proteins. Rahman and Lamsal [35] indicated that ultrasound-induced molecular motion, unfolding, and protein rearrangement primarily depend on the protein sources and ultrasound conditions. For instance, Hu et al. [43] noted that high-intensity ultrasound treatments did not alter the secondary structure of soybean β-conglycinin (7S) and glycinin (11S) fractions. Similarly, Byanju et al. [44] reported minimal changes in the secondary structures of soy and chickpea proteins when high-power ultrasound (3 and 5 W/mL) was applied. In both cases, the authors suggested that the discrepancies between their studies and others indicated that ultrasound treatment modifying the secondary structure of proteins might be attributed to differences in the proteins and sonication conditions used.

Therefore, US treatment can promote the unfolding of protein chains by breaking non-covalent bonds, leading to changes in the secondary structure of proteins, which depend on the protein type.

#### 2.1.3. Particle Size and Microstructure of Protein Aggregates

**Figure 3:**
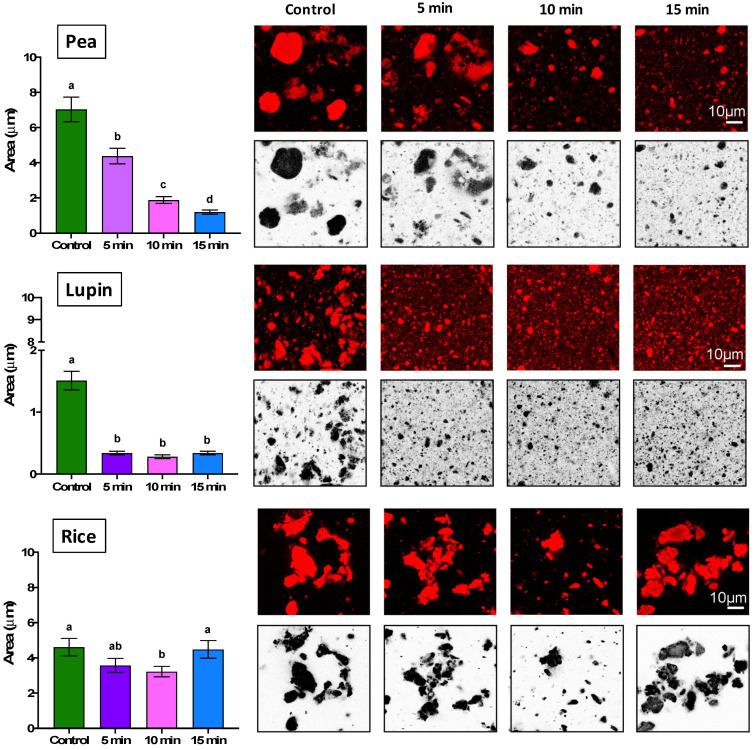
Effect of ultrasound treatment time on pea, lupin, and rice protein aggregates. Confocal microscopy images show protein aggregates from pea, lupin, and rice samples before (control) and after 5, 10, and 15 min of treatment. Red fluorescence highlights protein aggregates, while the same image is shown in grayscale for enhanced contrast. Different letters indicate significant differences (*p <* 0.05) between means for US treatment times.

The morphological and size changes in the plant proteins with different US times were visualized by confocal microscopy (Figure 3). First, a highly aggregated state (large and polydisperse irregularly shaped protein particles) was observed in all samples without US treatment, which can be attributed to the intermolecular aggregation that occurs during the processing to obtain these isolates [38,45]. In this sense, Schmitt and Wanasundara [46] indicated that commercial plant proteins exhibit oligomeric or aggregated states due to the various extraction processes affecting their techno-functional properties.

Regarding the native plant proteins (control samples), differences in the shape of the aggregates were observed (Figure 3). Pea protein displayed spherical particles with a uniform and ordered structure, while the morphology of the lupin and rice aggregates was irregular, with larger flocs, particularly in rice protein. Schmitt et al. [47] and Yang et al. [48] reported that extraction process conditions can induce structural rearrangements in proteins, leading to a mixture of randomly aggregated species. Furthermore, the larger and irregular flocs observed in rice protein may indicate a higher affinity for hydrophobic interactions or disulfide bond formation during extraction, which could contribute to its less uniform morphology [49]. On the other hand, the size of aggregates varied by protein type. The highest size values were found in pea protein (~7.03 µm), followed by rice and lupin protein (~4.61 and 1.51 µm, respectively) (Figure 3). The differences in the aggregate shape and size of the plant proteins could be due to their biological origin and the extraction and drying methods used in their production [6]. Considering biological origin, protein morphological properties are influenced mainly by their source, amino acid composition, and structural characteristics [48]. Pea proteins are rich in globular structures and hydrophobic regions, so they can exhibit larger aggregates due to strong hydrophobic interactions and flexibility [50]. Lupin proteins, with a higher proportion of hydrophilic residues, form smaller aggregates by reducing such interactions [51]. Rice proteins, dominated by glutelins and prolamins, stabilize aggregates through robust molecular interactions such as disulfide bonds [52]. On the other hand, extraction and drying methods (e.g., pH extremes, heat, or spray-drying) can further modify the aggregation behavior by affecting the protein structure and intermolecular forces, leading to changes in solubility, hydrophobicity, and functional properties [53].

When US treatment was applied, a significant decrease (*p* < 0.05) in the size of pea and lupin aggregates was observed (Figure 3). Regarding pea protein, the particle size decreased gradually with US time (4.38, 1.88, and 1.20 µm at 5, 10, and 15 min, respectively). However, the aggregate size of lupin protein decreased after 5 min of US treatment (from 1.51 to 0.34 µm), remaining constant until 15 min with values close to 0.3 µm. In contrast, the particle size of rice protein was only slightly affected by the US treatment time (between 4.61 and 4.48 µm) (Figure 3). O’Sullivan et al. [54] and Omura et al. [55] reported that the decrease in protein aggregate size after ultrasound treatment can be attributed to the disruption of the intermolecular interactions of protein aggregates. Specifically, cavitation forces break the hydrophobic and electrostatic interactions that stabilize untreated protein aggregates, decreasing protein size [54]. This phenomenon was clearly observed in pea and lupin proteins (Figure 3), where a notable reduction in aggregate size indicates a significant alteration in their three-dimensional structures, which can be attributed to the protein secondary structure changes observed by FT-IR analysis (Figure 2). However, the resistance of rice protein to ultrasound-induced changes suggests a different structural behavior. Rice protein exhibited minimal modifications in its secondary structure, indicating stronger or more rigid molecular interactions and less susceptibility to ultrasonic shear forces. Similar results were obtained by Igartúa et al. [22], where ultrasound treatment (5 min with 30/30 s on/off cycle and 100% amplitude) at neutral pH conditions did not generate significant changes in the particle size distribution of rice proteins. The authors concluded that the protein aggregation of rice proteins under neutral pH conditions is so intense that it cannot be broken by ultrasound treatment.

Therefore, pea and lupin proteins are more susceptible to unfolding and reductions in particle size by the effect of ultrasound treatment due to their less rigid aggregate structures. These structural differences may influence their techno-functional performance in gel-like food products.

### 2.2. Characterization of Plant Protein Gels

Based on the structural changes obtained for plant protein dispersion after treatment with different ultrasound times, heat-induced gels (90 °C for 60 min) were prepared using plant protein dispersion at 10% *w*/*w*.

#### 2.2.1. Gel Strength

**Figure 4:**
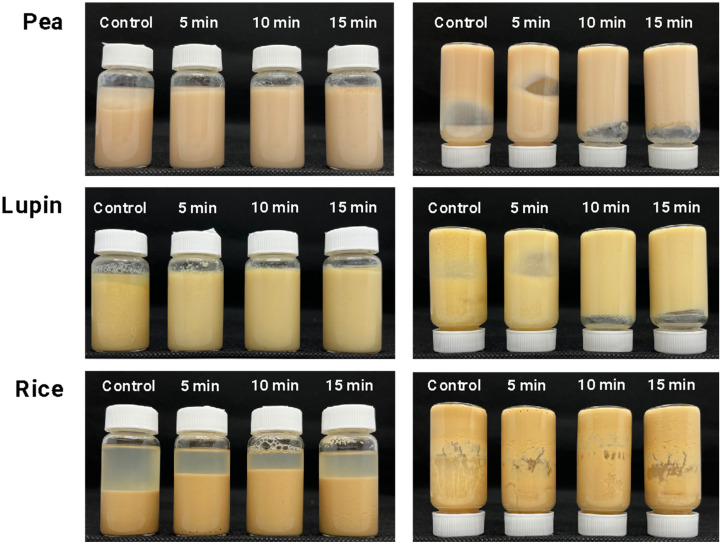
Photographs of gel formation of plant proteins treated with different times of ultrasound.

Gel strength is the most critical characteristic of protein-based gels, since it demonstrates the functionality of proteins and their ability to form a 3D network. Gel formation was characterized using photographs of the samples after heating treatment, as shown in Figure 4. First, no gel formation was observed in any plant protein controls (without US treatment). This is because protein gelation occurs through the unfolding of protein structures, the association–dissociation of protein subunits, and their subsequent aggregation to form a three-dimensional network [56]. As mentioned, the plant protein isolates (control samples) exhibited a highly aggregated structure (Figure 3), which could lead to partial unfolding during heat treatment. Also, this phenomenon can be attributed to the unavailability of the proteins’ hydrophobic regions to interact and develop a 3D network [48] resulting, therefore, in a poor gelation capacity.

**Figure 5:**
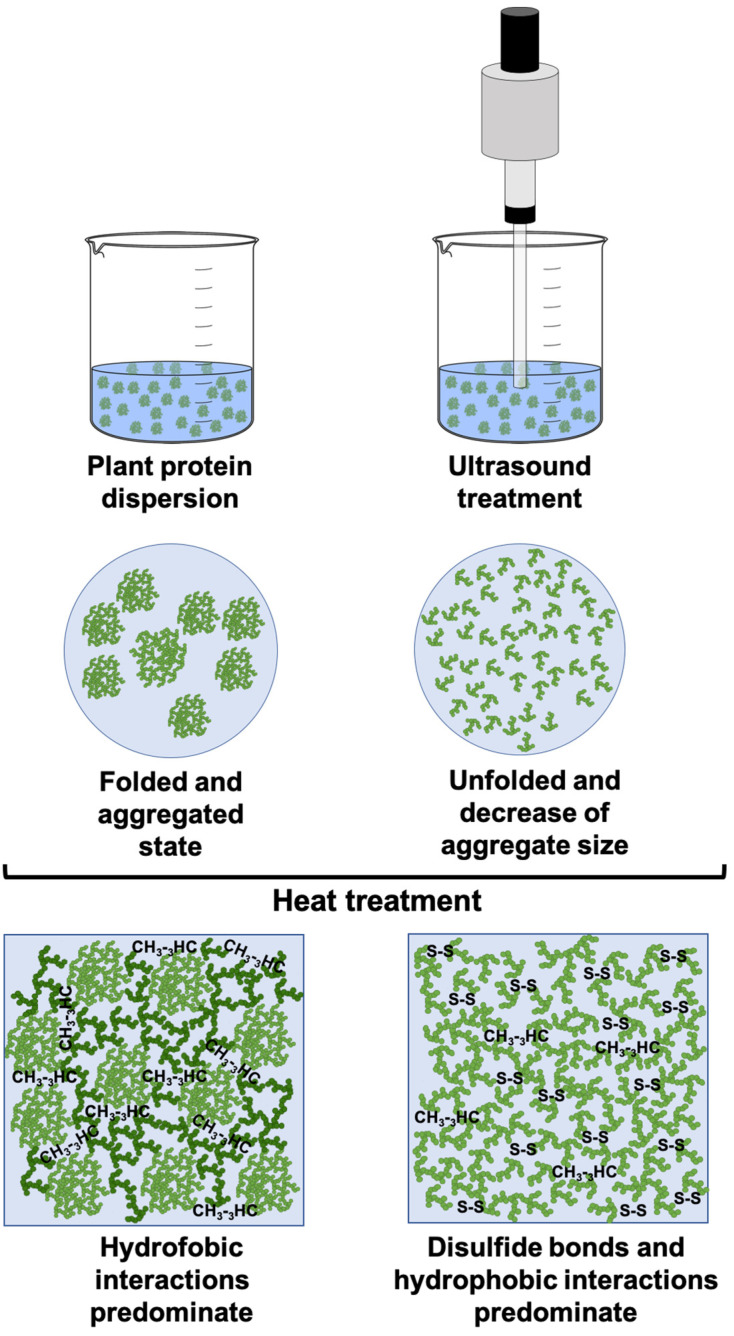
Schematic representation of gel formation from plant proteins with and without US treatment.

The results of gel strength indicated that ultrasound (US) treatment improved the gelling capacity of pea and lupin proteins, leading to soft gel after 5 and 10 min, respectively (Figure 4). This fact confirms that reducing plant protein particle size through US treatment (as shown in Figure 3) may facilitate the formation of a heat-induced gel network (Figure 5). An enhanced gel strength occurred because smaller particle sizes could unfold more easily, increasing surface hydrophobicity and the content of free sulfhydryl groups, which promote gel structure formation [57]. In this context, the observed changes in the secondary structures of pea and lupin proteins, characterized by an increased relative content of random coil and turn conformations (Figure 2), suggest a higher presence of disulfide bonds and hydrophobic interactions due to protein unfolding, which enhance gel network formation (Figure 5). Zhao et al. [39] also indicated that reducing particle size can lead to a tighter and more uniform network structure under thermal treatment, thereby enhancing the gel’s strength. However, no significant differences in the gel strength of pea and lupin proteins were observed by increasing the US treatment time, obtaining values of approximately 7 g-force (Table 1). These gel strength values are lower than those in other studies, e.g., 12.7 and 13 g-force for pea and lupin proteins, respectively [58,59], whereby the gels obtained in this work can be considered as weak and poorly structured gels. Despite this, these gels have potential applications in various food systems where a high gel strength is not required, such as desserts, puddings, mousses, or creamy spreads.

Finally, no changes in gel formation were observed when US treatment was applied to rice protein (Table 1 and Figure 4). This result is consistent with the fact that ultrasound treatment did not affect the structural properties of the rice protein. Rice proteins showed a significant aggregate state, which remained unchanged with an increasing ultrasound treatment time (Figure 3). Since protein–protein interactions play a crucial role in 3D network formation, the heat treatment was insufficient to unfold the rice protein aggregates, preventing these interactions, and, as a result, no gel formation occurred.

#### 2.2.2. Viscoelastic Properties

**Figure 6:**
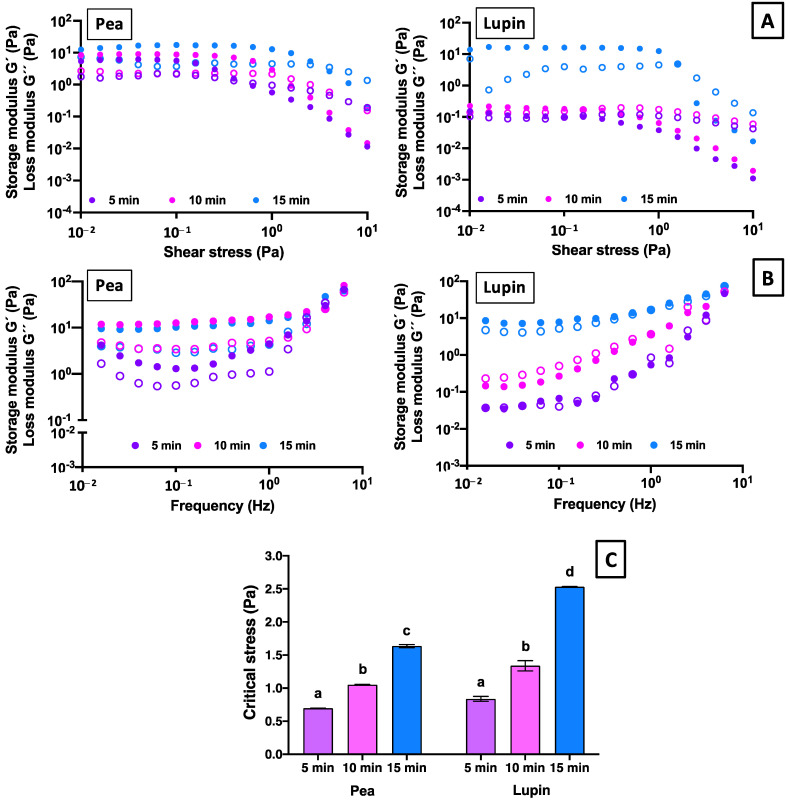
Stress sweeps (**A**), frequency sweeps (**B**), and critical stress (**C**) of gels prepared with plant proteins treated with different times of ultrasound. G′: filled symbols and G″: empty symbols. The viscoelastic properties of the pea and lupin controls and all rice protein samples were not determined due to the absence of gel formation. Different letters indicate significant differences (*p <* 0.05) between means for US treatment times.

Figure 6 presents the viscoelastic properties of the gels made with pea and lupin proteins, characterized by stress and frequency sweeps. The control samples of pea and lupin proteins and all samples of rice protein were not studied because they did not form gels.

First, stress sweeps were conducted to determine the stress range corresponding to the linear viscoelastic region (LVR). Figure 6A shows differences in the moduli (*G*′ and *G*″) values of the pea and lupin gels within the LVR of the samples tested, as well as the stress values at which the structural stability of the samples was compromised, marking the end of the LVR. This behavior was dependent on the US treatment time. Samples treated with 5 min of US exhibited a less extended LVR, where the *G*′ and *G*″ moduli values for pea and lupin proteins remained constant up to 0.69 and 0.83 Pa (critical stress), respectively (Figure 6C), indicating that the structures of the samples remained unaffected by oscillatory stress. In contrast, gels made from proteins treated with 10 and 15 min of US exhibited a broader linear viscoelastic region, with variations depending on the protein type (Figure 6A). For pea protein, the moduli values remained constant until 1.05 and 1.64 Pa for 10 and 15 min of US treatment, while the moduli values of lupin gels remained constant until 1.33 and 2.53 Pa for 10 and 15 min of US treatment, respectively (Figure 6C). The increase in the linear viscoelastic region of samples with longer US treatment times could be attributed to stronger protein–protein interactions. The US treatment likely enhances these interactions, making the gels more resistant to breaking under applied stress [60], where higher stress values are required to destroy the internal structure of protein gels [61]. Therefore, a more structured gel, capable of resisting greater stress, can be formed with an increasing US treatment time. Based on these results, a stress of 0.05 Pa was selected for the frequency assays.

The mechanical spectra show the variation in *G*′ and *G*″ across frequencies ranging from 0.01 to 10 Hz (Figure 6B). In general, most samples exhibited higher *G*′ values than *G*″ values, indicating gel-like behavior. However, the lupin-based gel treated for 5 min of US showed *G*″ > *G*′ values, revealing a fluid-like behavior. On the other hand, pea protein gels exhibited the highest *G*′ and *G*″ values compared to lupin samples (Figure 6B). These differences can be attributed to the protein composition, since lupin protein contains a higher number of sulfhydryl group bonds than pea protein (10 vs. 6 S-S bonds, respectively) [23]. The presence of sulfhydryl groups enhanced the thermal stability of the protein, which reduced its ability to form gels. Consequently, lupin protein formed weaker gels than pea protein under the same conditions. Additionally, the *G*′ and *G*″ values increased when the US time increased (Figure 6B), indicating that the network of the plant protein gels became stronger with US treatment. The US treatment increased protein aggregation and facilitated the formation of the gel network, especially at 15 min of treatment. Finally, lupin protein gels showed a strong frequency dependence in their *G*′ and *G*″ values, especially at a lower US time (5 min). This behavior could be due to the faster molecular mobility at higher frequencies, suggesting that the non-covalent bonds were the main forces for maintaining the gel network [61].

Regarding the viscoelastic parameters at 1 Hz, the ANOVA results showed that protein type, ultrasound time, and its interaction had significant effects (*p* < 0.05) on all gels’ viscoelastic parameters (Supplementary Materials, Table S1). For pea protein gels, the storage (*G*′) and loss (*G*″) moduli values at 1 Hz increased significantly with the US treatment time (Table 2). However, no significant differences (*p >* 0.05) in the *G*′ and *G*″ moduli values were observed after 10 min of US treatment. This behavior may be attributed to the reaggregation of pea protein chains at longer US treatment times (15 min), which reduces gelling capacity by weakening protein–protein and protein–water interactions [34]. Despite this, *G*′ remained greater than *G*″ for all pea protein samples, with similar (*p* > 0.05) loss tangent (tan δ = *G*″/*G*′) values, regardless of the US treatment time (Table 2). This suggests that the viscoelastic network properties of pea protein gels were not significantly influenced by US time. In addition, the tan δ values of pea protein gels were less than 1 (~0.3), confirming gel-like behavior, as the elastic component predominated [62]. Regarding lupin protein gels, the samples exhibited different behaviors according US treatment time. Gels made from proteins treated with 5 min of US showed a predominantly viscous behavior. However, after 10 and 15 min of US, the viscoelastic moduli of the lupin protein gels changed, where elastic moduli predominated more than viscous ones (Table 2), indicating a gel-like structure. These findings suggest that US treatment time influenced the viscoelastic properties of the lupin protein gels, as reflected in the tan δ values of 1.35, 0.73, and 0.35 for 5, 10, and 15 min of US treatment, respectively. A shorter US treatment resulted in an entangled dispersion, while a longer treatment led to a more structured gel network, exhibiting weak gel behavior.

## 3. Conclusions

Different ultrasound (US) treatment times were applied to enhance the gelling properties of pea, lupin, and rice protein isolates. FT-IR spectra and microstructure analysis indicated that US treatment induces structural modifications in the proteins through partial unfolding. This unfolding was related to the disruption of intermolecular interactions, increasing the exposure of hydrophobic regions, which also improved their gelling capacity. However, this effect depended on the protein type, since rice protein exhibits minimal structural alterations resulting from ultrasound (US) treatment compared to the pea and lupin proteins.

Regarding gelling properties, the US treatment significantly improves the gel-forming ability of the pea and lupin proteins. In addition, longer ultrasound times promoted stronger protein–protein interactions, resulting in firmer gels, which is particularly relevant for applications requiring a high gel strength and stability, such as dairy and fish analogs. Conversely, the gelling properties of rice protein remained unaffected by ultrasound treatment, presenting a challenge for its broader application in food systems.

From a practical perspective, these findings demonstrate that ultrasound treatment (5–15 min) can be used to tailor the gelling capability of plant proteins for specific food applications. For example, pea and lupin proteins treated with ultrasound showed great potential for use in plant-based analog foods, such as meat substitutes, dairy alternatives, and high-protein gel-type snacks. Future research could focus on optimizing ultrasound parameters and exploring other protein sources to expand the range of functional ingredients available for plant-based food innovations.

## 4. Materials and Methods

### 4.1. Materials

Samples were prepared with isolates of pea protein (Nutralys, Roquette, Pas-de-Calais, Lestrem, France; 84% protein), lupin protein (UltraProt, CGNA, Temuco, Araucania, Chile; 90% protein), and rice protein (Angeloni^®^ Organics, Las Condes, Santiago, Chile; 90% protein). Rhodamine B was purchased from Sigma-Aldrich (Burlington, MA, USA), and other chemicals and reactants (analytical grade) were purchased from Winkler Ltda. (Lampa, Santiago, Chile). Also, deionized water (28.5 μs of conductivity) obtained from a reverse osmosis system (VigaFlow S.A., Colina, Santiago, Chile) was used for the sample preparation and analysis.

### 4.2. Ultrasound Treatment

First, plant protein dispersions (10% *w*/*w*) were prepared by adding pea, lupin, and rice protein isolates into 10 mM buffer phosphate (pH 7) and stirred (MS-H280-Pro, DLab, Shunyi District, Beijing, China) at room temperature (25 ± 1 °C) for 3 h. Then, plant protein dispersions were treated with different ultrasound (US) times (5, 10, and 15 min) using an ultrasonic processor (VCX500, Sonics, Orlando, FL, USA) equipped with a ½-inch probe (13 mm in diameter) with the following treatment conditions: 20 kHz, 60% amplitude, and 30 and 30 s work and rest intervals, respectively. US times were selected according to previous results in our laboratory. The treatment was carried out in an ice-water bath to prevent the sample from overheating. In addition, three control samples (one for each protein type) were prepared without ultrasound treatment. All protein dispersions were freeze-dried (FD5508, Ilshin, Sasang-gu, Busan, Republic of Korea), vacuum-packed, and stored at 4 °C for further characterization. Finally, the protein contents of the samples were 73–76%, 68–74%, and 71–73% for pea, lupin, and rice protein, respectively.

### 4.3. Characterization of Freeze-Dried Plant Protein

#### 4.3.1. Optical Properties

The optical properties of the freeze-dried plant proteins, both US-treated and control samples, were evaluated using a colorimeter (CR-410, Konica Minolta, Nishi-ku, Osaka, Japan), previously calibrated with a standard plate (L*: 93.46, a*: 0.42, and b*: 4.08). Each sample (~10 g) was deposited in a Petri dish (60 mm in diameter), and the whiteness index (WI) was calculated from CIELab parameters (L*: brightness, a*: redness to greenness, and b*: blueness to yellowness) using Equation (1).

(1) $$WI\left(\%\right)=100-\sqrt{{\left(100-L^{*}\right)}^{2}+a^{*2}+b^{*2}}$$

#### 4.3.2. Fourier Transform Infrared (FT-IR) Spectroscopy

The secondary structure of all freeze-dried plant proteins was analyzed using an FT-IR spectrometer equipped with an attenuated total reflectance (ATR) unit (Diamond Two, Perkin Elmer, Beaconsfield, Buckinghamshire, UK). For that, pellet freeze-dry samples (~1 mg) were transferred to the FT-IR unit and scanned at a 4000–400 cm^−1^ wavelength range with 64 scans at a 1 cm^−1^ resolution. Calibration was performed using a background spectrum recorded from the clean and empty cell at room temperature (25 °C). The spectra obtained were analyzed using Spectragryph software (F. Menges, Spectragryph—optical spectroscopy software, version 1–202, http://www.effemm2.de/spectragryph/, accessed on 28 October 2024) to determine the secondary structure (α-helix, β-sheet, β-turn, and random coil) changes in samples with ultrasound time.

#### 4.3.3. Microstructure

The microstructures of the plant proteins were analyzed using a Confocal Laser Scanning Microscope (CLSM) (TCS SP8, Leica Microsystems, Wetzlar, Hesse, Germany) at room temperature (25 °C). For that, 900 μL of freeze-dried protein rehydrate at 10% *w*/*w* was mixed with 100 μL of 0.001% *w*/*w* Rhodamine B (as a final concentration in the mixture). Then, 10 μL of the mixture was placed on a slide, covered with a coverslip, and observed under a microscope at 63× magnification using silicone oil and Zoom 1. The excitation and emission wavelengths were 555 and 630 nm, respectively. Image J software was used to analyze the CLSM images (1024 × 1024 pixels) (version 1.x, https://imagej.net/, accessed on 2 December 2024).

### 4.4. Preparation and Characterization of Plant Protein Gels

#### 4.4.1. Preparation of Plant Protein Gels

The US-treated and control freeze-dried samples were dispersed in distilled water (10% *w*/*w* protein) for gel preparation. The dispersions were stirred for 3 h at room temperature (25 ± 1 °C) and then placed in glass vials with screw caps (20 mL capacity). The samples were heated at 90 ± 1 °C for 60 min in a thermoregulated water bath (B-100, Buchi, Flawil, Postfach, Switzerland). Then, the sol obtained was cooled at room temperature and refrigerated overnight (4 °C) to induce gelation.

#### 4.4.2. Gel Strength

Gel strength (g) was measured using a texture analyzer (TA-XT express, Stable Micro Systems Ltd., Godalming, UK) at room temperature. Gels (25 mm diameter and 40 mm height) were compressed with a 5 mm stainless steel cylinder probe (P/0.5R, ½” diameter cylinder) into a vial using a trigger force of 0.0098 N, test speed of 0.5 mm s^−1^, and test distance (depth reached by the probe) of 0.5 mm.

#### 4.4.3. Viscoelastic Properties

The viscoelasticity of plant protein dispersions was measured using a rheometer (Discovery HR-2, TA Instruments, New Castle, DE, USA) with parallel-plate geometries (diameter: 25 mm; gap: 1 mm). Measurements were conducted at 25 ± 1 °C controlled by a thermostat system (ThermoCube 200–500, Solid state cooling systems, New Castle, DE, USA). After loading the sample in the rheometer, it was allowed to stand for 10 min to stabilize and reach the test temperature. First, stress sweeps between 0.01 and 100 Pa, at a frequency of 0.1 Hz, were made to determine the linear viscoelasticity range (LVR). Then, frequency sweeps at 0.05 Pa were performed from 0.01 to 10 Hz. Finally, the values of storage modulus (*G*′), loss modulus (*G*″), and loss tangent angle (tan δ) at 1 Hz were calculated to compare the viscoelastic properties among different protein gels. Measurements were made in duplicate.

### 4.5. Statistical Analysis

All measurements were performed in duplicate at a minimum, and the instrumental data obtained were expressed with mean ± standard deviation. The statistical differences between protein type and ultrasound time were determined by two-way ANOVA (Analysis of Variance) with Fisher’s LSD multi-comparison test with a significance level of *α =* 0.05 through XLSTAT© software (version 2024.3, Denver, CO, USA).

## Figures and Tables

**Table 1:** Gel strength of gels based on plant proteins treated at different times of ultrasound.

Ultrasound Time	Gel Strength (g-Force)		
	Pea	Lupin	Rice
Control	n.d	n.d	n.d
5 min	7.00 ± 0.07 ^a^	n.d	n.d
10 min	7.05 ± 0.52 ^a^	6.77 ± 0.55 ^a^	n.d
15 min	7.77 ± 0.29 ^a^	6.97 ± 0.42 ^a^	n.d

Note: n.d means that gel strength was not determined since no gel was formed. Different letters in the same column indicate significant differences (*p <* 0.05) between means for US treatment times.

**Table 2:** Mechanical parameters at 1 Hz of plant proteins treated with different times of ultrasound.

Protein Type	Ultrasound Time	Storage Modulus (Pa)	Loss Modulus (Pa)	tan δ (-)
Pea	5 min	5.18 ± 1.04 ^b^	1.59 ± 0.65 ^bc^	0.30 ± 0.07 ^c^
	10 min	16.12 ± 0.98 ^a^	6.47 ± 1.86 ^a^	0.30 ± 0.01 ^c^
	15 min	13.48 ± 1.00 ^a^	4.15 ± 0.22 ^ab^	0.32 ± 0.03 ^c^
Lupin	5 min	0.79 ± 0.22 ^c^	1.01 ± 0.23 ^c^	1.35 ± 0.32 ^a^
	10 min	2.96 ± 1.05 ^bc^	0.73 ± 0.10 ^c^	0.67 ± 0.02 ^b^
	15 min	14.34 ± 3.60 ^a^	5.14 ± 2.20 ^a^	0.35 ± 0.06 ^bc^

Note: Different letters in the same column indicate significant differences (*p* < 0.05) between means for US treatment times. The mechanical properties of the pea and lupin controls and all rice protein samples were not determined due to the absence of gel formation. tan δ (*G*″/*G*′) corresponds to loss angle and is dimensionless.

## Data Availability

Data will be made available on request.

## Supplementary Materials

The following supporting information can be downloaded at: https://www.mdpi.com/article/10.3390/gels11040270/s1, Table S1: Two-way ANOVA of rheological parameters of gels prepared with plant proteins treated with different times of ultrasound.
