# Supplementary material for: Effect of Ultrasound Time on Structural and Gelling Properties of Pea, Lupin, and Rice Proteins
Source: Gels. 2025 Apr 4;11(4):270. doi: 10.3390/gels11040270 (PMC12026612; doi:10.3390/gels11040270)
Supplement: Supplementary file 1 [file gels-11-00270-s001.zip › gels-3528952-supplementary.pdf]

Supplementary Materials

Table S1. Two-way ANOVA of rheological parameters of gels prepared with plant-proteins treated with different times of ultrasound.

			Rheological parameters		
			Storage modulus (Pa)	Loss modulus (Pa)	Tan **δ** (-)
Main effects	A: Protein type	***F-value***	31.81	6.43	36.77
		p-value	0.001	0.044	0.001
	B: Ultrasound time	***F-value***	38.75	7.96	13.29
		p-value	<0.001	0.020	0.006
Interaction	A x B	***F-value***	19.81	8.44	14.25
		p-value	0.002	0.018	0.005
